# Thyroid hormones and prognosis in adults with status epilepticus: a retrospective study

**DOI:** 10.3389/fendo.2024.1452299

**Published:** 2024-11-08

**Authors:** Jie Fu, Xiu Chen, Jinglun Li, Lilei Peng

**Affiliations:** ^1^ Department of Neurology, The Affiliated Hospital of Southwest Medical University, Luzhou, China; ^2^ Department of Neurosurgery, The Affiliated Hospital of Southwest Medical University, Luzhou, China

**Keywords:** status epilepticus, thyroid hormones, free triiodothyronine, low T3 syndrome, prognosis

## Abstract

**Objectives:**

Thyroid hormone levels have been indicated to be associated with the functional outcome in critical illness. However, the studies on thyroid hormones and status epilepticus (SE) are rare. This study aimed to evaluate the predictive value of serum thyroid hormone levels on admission for unfavorable outcome in adult patients with SE.

**Methods:**

We investigated and validated the predictive value of serum thyroid hormone levels on admission for the prognosis of adult SE patients. We extracted the clinical information and outcomes of patients. Modified Rankin scale (mRS) scores were applied to assess the patients’ functional outcome, and mortality at 30 days after SE onset was identified. Serum levels of thyroid hormones including free thyroxin (FT4), free triiodothyronine (FT3) and thyroid-stimulating hormone (TSH) were detected on admission.

**Results:**

We first analyzed the discovery cohort of 87 patients with SE. We found that 35.6% (31/87) of the patients had a poor outcome at discharge, and 18.4% (16/87) of the patients died during hospital stay and at 30-day follow up. The serum FT3 levels in the non-survivors group were significantly lower than those in the survivors group. Low T3 syndrome occurred in 29.9% (26/87) of SE cases and patients with low T3 syndrome were more likely to have unfavorable outcomes. Furthermore, we observed similar results in the external cohort, which validated our findings.

**Conclusions:**

Serum FT3 levels measured on admission are independently associated with 30-day mortality in SE patients. Additionally, low T3 syndrome may be a promising candidate for predicting SE prognosis.

## Introduction

Status epilepticus (SE) is a life-threatening neurological emergency with considerable mortality and morbidity (1). The mortality rate of SE is reported to range from 5 to 46% (2). Furthermore, survivors of SE may have long-term adverse sequelae (3). Predicting the prognosis of SE is challenging due to the heterogeneous etiologies and diverse clinical manifestations. Prior studies have proposed some scales, such as status epilepticus severity score (STESS) and epidemiology-based mortality score in status epilepticus (EMSE), to help clinicians to predict SE outcome. STESS scale consists of four variables: history of seizures, age, seizure type and consciousness level, which can be used to assess the risk of mortality early in SE (4). Thereafter, the EMSE scale, a clinical-electrophysiological tool, was reported to predict mortality in SE, which applied a complicated scoring system with a combination of etiology, age, comorbidity, and characteristics on electroencephalography (EEG) (5). However, these scales have some limitations under specific conditions. SE patients with consciousness impairment may not be able to provide the previous history of epilepsy, especially when their relatives are not present. Additionally, EEG examination is not available for every SE patient in every region. An ideal tool for predicting SE outcome should be simple and easily available markers, which could be useful to help clinicians to identify SE patients with high risk of poor prognosis, and thus early and adequate interventions could be taken to improve these patients’ functional outcomes.

Thyroid hormones are crucial for brain development and maturation, and also play an important role in maintaining brain functions (6). The thyroid gland produces and releases thyroxin (T4) and triiodothyronine (T3), which is modulated by thyroid-stimulating hormone (TSH) (7). Both T4 and T3 have a bound form and a free form, and the free form is bioactive (8). Critical illness may lead to a reduced peripheral conversion of free T4 (FT4) to free T3 (FT3), which contributes to the development of low T3 syndrome (decreased serum T3, low or normal serum T4, and normal TSH levels) (7, 9). Previous studies have indicated that thyroid hormone alterations as well as low T3 syndrome are associated with the prognosis of various neurological diseases, such as ischemic stroke (10) and autoimmune encephalitis (9). Of note, thyroid hormones have been indicated to be involved in the pathogenesis of epilepsy (11). Thyroid hormones affect neurotransmission, as well as regulate the development and function of γ-aminobutyric acid (GABAergic) interneurons, and thus impact inhibitory and excitatory neuronal circuits, further participating in triggering or sustaining epileptic activity (12, 13). However, the prognostic value of thyroid hormones in SE patients remains unclear.

In the present study, we aimed to explore the predictive value of serum levels of thyroid hormones for unfavorable outcome at discharge as determined by the modified Rankin scale (mRS) score as well as 30-day mortality in adult patients with SE. Furthermore, we sought to investigate whether low T3 syndrome could predict the prognosis of adult patients with SE.

## Methods

### Study design and ethics

Two independent cohorts were included in the present study. Cohort 1 served as the discovery cohort, and we reviewed the medical records of patients with SE admitted to the affiliated hospital of Southwest Medical University from January 2020 to December 2023. Cohort 2 was an external validation cohort, which included a dataset of SE patients from the affiliated Traditional Chinese Medicine hospital of Southwest Medical University between January 2021 and December 2023. The present study was approved by the ethics committees of the affiliated hospital of Southwest Medical University and the affiliated Traditional Chinese Medicine hospital of Southwest Medical University. Informed consent was obtained from all patients or family members.

### Definitions and criteria

SE patients aged at least 18 years old without previous history of thyroid diseases were included. SE was defined as prolonged clinical and/or electrographic seizure activity which lasted for more than five minutes, or recurrent seizure activity with no complete functional recovery in between (14). Refractory SE was defined as no response to first-line and second-line antiepileptic drugs (AEDs) (15). Super-refractory SE is that seizure activity continues for 24 hours or longer after the initiation of anesthetic treatment (16). Patients with SE caused by hypoxic-ischemic encephalopathy after cardiac arrest were excluded. SE etiology was classified into four groups: acute symptomatic SE, remote symptomatic SE, symptomatic SE due to progressive brain disorders, or unprovoked SE of unknown etiology, which was suggested by the International League Against Epilepsy (ILAE) (17). Status epilepticus severity score (STESS) scale consists of four variables: history of seizures, age, seizure type and consciousness level (4). Low T3 syndrome was defined as serum FT3 below the lower limit of the reference interval along with normal TSH levels (9). Clinical outcomes at discharge were evaluated using the mRS score. An mRS score of less than 3 was regarded a good outcome, while an mRS score of equal to or above 3 (including death) was regarded a poor outcome.

### Data collection

Clinical information was collected, including age; sex; etiology of SE; previous history of seizures; STESS at SE onset; SE duration; the number of AEDs. Data of laboratory tests including white blood cell count, neutrophil count, lymphocyte count, monocyte count, red blood cell count, red cell distribution width, platelet count, albumin, FT3, FT4 and TSH within 24 hours after admission were collected. Data of outcomes included mRS scores at hospital discharge and mortality at 30-day follow up after SE onset. The follow-up information was obtained from the hospital records or by interviewing (in person or by telephone) the patients and their relatives.

### Statistical analysis

The Shapiro-Wilk test was conducted to distinguish between normal and non-normal distributions. Continuous variables are presented as means (standard deviation) or medians (interquartile range, IQR) for normally distributed and skewly distributed variables, respectively, while categorical variables were described as frequency and percentage. For comparative analysis of three or more groups, analysis of variance or Kruskal-Wallis test was used for normally distributed and skewly distributed data, respectively. Univariate analyses were performed by using the t-test, the Mann-Whitney U-test and the chi-square (χ^2^) test. The t-test or Mann-Whitney U-test were employed to compare continuous data, and the chi-square (χ^2^) test was applied to compare categorical variables. Variables with *P* < 0.10 in the univariate analysis were screened to enter into the multivariate logistic regression model. Multicollinearity was evaluated with the variance inflation factor (VIF). The variable selected for subsequent multivariate analysis was tolerance > 0.1 and VIF < 5. Receiver operating characteristic (ROC) curve analysis was used to assess the predictive value of FT3 for 30-day mortality in SE patients. The cutoff point of FT3 was set by the Youden index. All statistical analyses were carried out by using GraphPad Prism 9.0 and SPSS 26.0 softwares, and *P* values < 0.05 were regarded significant.

## Results

### Baseline characteristics of patients with SE

**Figure 1:**
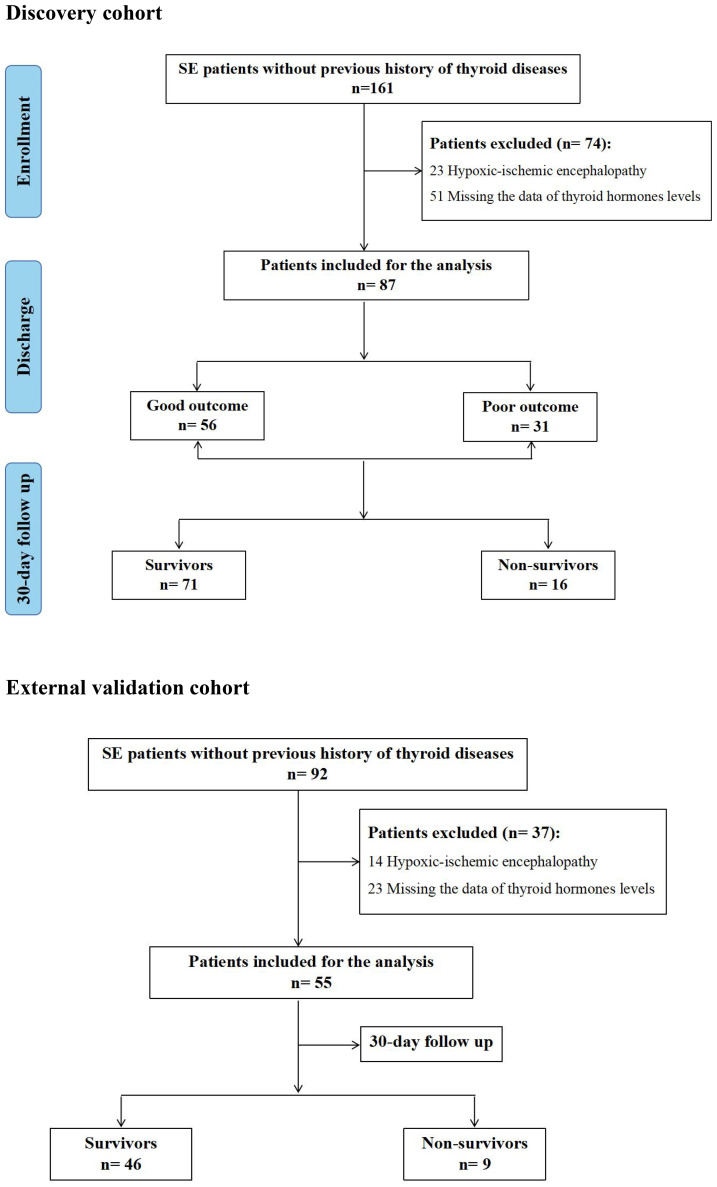
Flowchart of study patients.

In the Discovery cohort, Out of 161 patients with SE, 23 patients with hypoxic-ischemic encephalopathy and 51 patients without measurements of thyroid hormones within 24 hours after admission were ruled out. Of the 87 SE patients included in the analysis, 31 patients (35.6%) suffered from poor outcomes at discharge, and 16 patients (18.4%) died during hospital stay and at 30-day follow up after SE onset. Additionally, 26 of the 87 patients (29.9%) met the diagnostic criteria of low T3 syndrome. In the external validation cohort, a total of 55 SE patients meeting the inclusion criteria were included. Out of 55 SE patients, 9 patients (16.4%) died during hospital stay and at 30-day follow up after SE onset, and 15 patients (27.3%) met the diagnostic criteria of low T3 syndrome. The flowchart of the study patients was shown in **Figure 1**.

### Thyroid hormones in SE patients

The results of thyroid hormones testing among SE patients with different etiologies and different classes of SE severity were summarized in **Tables 1**, **2**, respectively. No significant difference was observed in serum FT3, FT4 and TSH levels among SE patients with different causes (all *P* > 0.05, **Table 1**). In addition, serum TSH and FT4 levels were comparable among patients with different classes of SE severity, while FT3 levels were remarkably decreased as the SE severity increased, and patients with super-refractory SE had the lowest FT3 levels (*P* = 0.012, **Table 2**).

**Table 1:** Thyroid hormones in SE patients with different etiologies.

Variable	Causes of SE				
	Stroke (n=11)	Infection (n=11)	Metabolic disorders (n=5)	Other or unknown causes (n=60)	*P*
FT3 (pg/mL), mean (SD)	2.01 (0.47)	1.88 (0.56)	2.53 (0.49)	1.97 (0.59)	0.196
FT4 (ng/dL), mean (SD)	1.18 (0.35)	1.30 (0.45)	1.15 (0.22)	1.14 (0.31)	0.524
TSH (mIU/L), median (IQR)	1.69 (0.52-3.17)	0.82 (0.53-1.99)	1.16 (0.80-1.28)	1.20 (0.67-2.53)	0.720

SE, status epilepticus; FT3, free triiodothyronine; FT4, free thyroxin; TSH, thyroid-stimulating hormone; IQR, interquartile range; SD, standard deviation.

**Table 2:** Thyroid hormones in SE patients with different classes of severity.

Variable	SE^a^ (n=42)	Refractory SE^b^ (n=37)	Super-refractory SE (n=8)	*P*
FT3 (pg/mL), mean (SD)	2.12 (0.57)	1.96 (0.58)	1.46 (0.30)	0.012
FT4 (ng/dL), median (IQR)	1.14 (0.94-1.32)	1.18 (0.93-1.34)	0.99 (0.91-1.06)	0.293
TSH (mIU/L), median (IQR)	1.56 (0.77-2.58)	1.11 (0.64-2.47)	0.57 (0.31-1.53)	0.134

SE, status epilepticus; FT3, free triiodothyronine; FT4, free thyroxin; TSH, thyroid-stimulating hormone; IQR, interquartile range; SD, standard deviation.

a SE patients except for refractory SE.

b Refractory SE except for super-refractory SE.

### Thyroid hormones and poor outcome at discharge

Univariable comparisons of clinical characteristics and data of laboratory tests at admission between SE patients with good outcome and poor outcome at discharge were shown in **Table 3**. SE patients with poor outcome at discharge were older as compared to patients with good outcome (patients with poor outcome had median age 60 years, IQR 49-69 versus median age 47 years, IQR 28-58 in patients with good outcome; *P* = 0.006). Moreover, SE patients with poor outcome had significantly greater proportions of super-refractory SE and abnormal thyroid function, higher baseline red cell distribution width and STESS, as well as lower baseline red blood cell count, platelet count and albumin.

**Table 3:** Univariate analysis of clinical characteristics and laboratory data between SE patients with good or poor outcomes at discharge.

Variable	Good-outcome (n=56)	Poor-outcome (n=31)	*P*
Male (n, %)	32 (57.1)	18 (58.1)	0.934
Age, years, median (IQR)	47 (28–58)	60 (49-69)	0.006
SE etiology grouped according to the ILAE (n, %)			0.559
Acute symptomatic seizures	18 (32.1)	13 (41.9)	
Remote symptomatic unprovoked seizures	8 (14.3)	5 (16.1)	
Symptomatic seizures due to progressive CNS disorders	9 (16.1)	2 (6.5)	
Seizures of unknown etiology	21 (37.5)	11 (35.5)	
Stroke etiology of SE (n, %)	6 (10.7)	5 (16.1)	0.696
Infectious etiology of SE (n, %)	7 (12.5)	4 (12.9)	0.778
Metabolic etiology of SE (n, %)	3 (5.4)	2 (6.5)	0.787
No history of seizures (n, %)	40 (71.4)	23 (74.2)	0.782
Refractory SE (n, %)	27 (48.2)	18 (58.1)	0.379
Super-refractory SE (n, %)	2 (3.6)	6 (19.4)	0.040
STESS at SE onset, median (IQR)	2 (2-2)	3 (2-4)	0.002
SE duration, days, median (IQR)	1 (1-3)	2 (1-3)	0.107
AEDs, median (IQR)	2 (2-4)	3 (3-4)	0.115
RBC (x10^6^/μL), mean (SD)	4.54 (0.59)	4.17 (0.77)	0.014
WBC (x10^3^/μL), median (IQR)	9.68 (6.70-11.22)	10.52 (6.66-14.34)	0.168
NEU (x10^3^/μL), median (IQR)	6.81 (4.31-8.86)	7.29 (5.22-10.56)	0.321
LYM (x10^3^/μL), median (IQR)	1.26 (0.96-1.80)	1.24 (0.84-1.67)	0.527
MON (x10^3^/μL), median (IQR)	0.48 (0.35-0.63)	0.59 (0.37-0.75)	0.167
PLT (x10^3^/μL), median (IQR)	222 (177-288)	188 (143-246)	0.039
RDW (fL), mean (SD)	42.2 (3.4)	45.7 (4.7)	<0.001
ALB (g/L), median (IQR)	43.9 (40.3-46.9)	41.5 (34.7-42.9)	0.001
Abnormal thyroid function (n, %)	17 (30.4)	24 (77.4)	<0.001
FT3 (pg/mL), median (IQR)	2.18 (1.95-2.43)	1.61 (1.36-1.86)	<0.001
FT4 (ng/dL), median (IQR)	1.12 (0.91-1.32)	1.14 (1.00-1.33)	0.471
TSH (mIU/L), median (IQR)	1.36 (0.75-2.65)	0.82 (0.38-1.79)	0.018

SE, status epilepticus; IQR, interquartile range; ILAE, International League Against Epilepsy; CNS, central nervous system; STESS, status epilepticus severity score; AEDs, antiepileptic drugs; SD, standard deviation; RBC, red blood cell count; RDW, red cell distribution width; WBC white blood cell count; NEU, neutrophil count; LYM, lymphocyte count; MON, monocyte count; PLT, platelet count; ALB, albumin; FT4, free thyroxin; FT3, free triiodothyronine; TSH, thyroid-stimulating hormone.

Serum levels of FT3 and TSH were remarkably lower in patients with poor outcome than in patients with good outcome (**Table 3**). Serum FT4 levels were higher in patients with poor outcome, but the difference was not statistically significant (*P* = 0.471). Furthermore, a multivariate logistic regression analysis was conducted including age, super-refractory SE, abnormal thyroid function, STESS, red blood cell count, red cell distribution width, platelet count, albumin, FT3 and TSH (**Table 4**). These variables were selected as they were observed to be significantly different between patients with good outcome and poor outcome at discharge in the univariable analysis above (all *P* < 0.10, **Table 3**). The multivariate analysis indicated no significant association between serum FT3 and TSH levels and poor outcome (FT3: odds ratio (OR) = 0.659; 95% confidence interval, 0.146-2.973; *P* = 0.587; TSH: OR = 0.552; 95% confidence interval, 0.299-1.020; *P* = 0.058).

**Table 4:** Multivariate analysis of predictors for SE patients with poor outcome at discharge.

Variable	OR	95% CI	*P*
Age	1.005	0.963-1.050	0.809
RBC	1.045	0.318-3.432	0.942
PLT	0.993	0.984-1.002	0.111
RDW	1.252	1.029-1.522	0.025
ALB	0.901	0.785-1.033	0.136
FT3	0.659	0.146-2.973	0.587
TSH	0.552	0.299-1.020	0.058
STESS	1.670	0.769-3.629	0.195
Super-refractory SE	3.227	0.256-40.634	0.365
Abnormal thyroid function	3.050	0.617-15.068	0.171

SE, status epilepticus; OR, odds ratio; CI, confidence interval; RBC, red blood cell count; RDW, red cell distribution width; PLT, platelet count; ALB, albumin; FT3, free triiodothyronine; TSH, thyroid-stimulating hormone; STESS, status epilepticus severity score.

### Thyroid hormones and mortality at 30-day follow up

Univariable comparisons of clinical characteristics and data of laboratory tests at admission between survivors and non-survivors were shown in **Table 5**. Non-survivors had significantly greater proportions of refractory SE and abnormal thyroid function, higher baseline neutrophil count and STESS, and lower albumin levels compared to survivors. Serum levels of FT3 and TSH were remarkably lower in non-survivors than in survivors. Serum FT4 levels were lower in non-survivors, but the difference was not statistically significant (*P* = 0.132). SE etiology, refractory SE, super-refractory SE, abnormal thyroid function, STESS, albumin, FT3 and TSH were screened for the further multivariate logistic regression analysis (**Table 6**) as these variables were associated with 30-day mortality in the univariate analysis (all *P* < 0.10, **Table 5**). The multivariate analysis demonstrated that FT3 was an independent predictor of 30-day mortality (OR = 0.077; 95% confidence interval, 0.009-0.695; *P* = 0.022).

**Table 5:** Univariate analysis of clinical characteristics and laboratory data between survivors and non-survivors.

Variable	Survivors (n=71)	Non-survivors (n=16)	*P*
Male (n, %)	42 (59.2)	8 (50.0)	0.503
Age, years, median (IQR)	51 (29-61)	59 (47-68)	0.129
SE etiology grouped according to the ILAE (n, %)			0.053
Acute symptomatic seizures	21 (29.6)	10 (62.5)	
Remote symptomatic unprovoked seizures	13 (18.3)	0 (0)	
Symptomatic seizures due to progressive CNS disorders	10 (14.1)	1 (6.3)	
Seizures of unknown etiology	27 (38.0)	5 (31.2)	
Stroke etiology of SE (n, %)	7 (9.9)	4 (25.0)	0.219
Infectious etiology of SE (n, %)	7 (9.9)	4 (25.0)	0.219
Metabolic etiology of SE (n, %)	4 (5.6)	1 (6.3)	0.618
No history of seizures (n, %)	49 (69.0)	14 (87.5)	0.236
Refractory SE (n, %)	33 (46.5)	12 (75.0)	0.039
Super-refractory SE (n, %)	4 (5.6)	4 (25)	0.052
STESS at SE onset, median (IQR)	2 (2-3)	3 (2-4)	0.016
SE duration, days, median (IQR)	1 (1-3)	1.5 (1-4)	0.687
AEDs, median (IQR)	3 (2-4)	3 (3-4)	0.198
RBC (x10^6^/μL), mean (SD)	4.43 (0.66)	4.31 (0.75)	0.515
WBC (x10^3^/μL), median (IQR)	9.63 (6.49-11.22)	12.31 (8.04-13.92)	0.053
NEU (x10^3^/μL), median (IQR)	6.42 (4.14-8.97)	9.41 (6.54-12.41)	0.012
LYM (x10^3^/μL), median (IQR)	1.26 (0.96-1.79)	1.20 (0.72-1.81)	0.455
MON (x10^3^/μL), median (IQR)	0.48 (0.35-0.71)	0.57 (0.39-0.75)	0.305
PLT (x10^3^/μL), median (IQR)	209 (165-280)	219 (151-257)	0.631
RDW (fL), median (IQR)	43.2 (40.9-45.6)	43.9 (39.8-49.3)	0.334
ALB (g/L), mean (SD)	42.6 (5.4)	38.6 (7.4)	0.016
Abnormal thyroid function (n, %)	29 (40.8)	12 (75.0)	0.013
FT3 (pg/mL), mean (SD)	2.11 (0.51)	1.48 (0.60)	<0.001
FT4 (ng/dL), median (IQR)	1.15 (0.94-1.34)	1.02 (0.91-1.09)	0.132
TSH (mIU/L), median (IQR)	1.32 (0.72-2.56)	0.59 (0.31-1.51)	0.031

SE, status epilepticus; IQR, interquartile range; ILAE, International League Against Epilepsy; CNS, central nervous system; STESS, status epilepticus severity score; AEDs, antiepileptic drugs; SD, standard deviation; RBC, red blood cell count; RDW, red cell distribution width; WBC white blood cell count; NEU, neutrophil count; LYM, lymphocyte count; MON, monocyte count; PLT, platelet count; ALB, albumin; FT4, free thyroxin; FT3, free triiodothyronine; TSH, thyroid-stimulating hormone.

**Table 6:** Multivariate analysis of predictors for 30-day mortality in SE patients.

Variable	OR	95% CI	*P*
SE etiology			
Acute symptomatic seizures	Reference	–	–
Remote symptomatic unprovoked seizures	0.000	0.000	0.998
Symptomatic seizures due to progressive CNS disorders	1.063	0.077-14.718	0.964
Seizures of unknown etiology	0.121	0.017-0.831	0.032
ALB	0.947	0.828-1.084	0.433
FT3	0.077	0.009-0.695	0.022
TSH	0.618	0.333-1.148	0.128
Refractory SE	3.003	0.554-16.270	0.202
Super-refractory SE	2.320	0.237-22.754	0.470
STESS	2.083	1.001-4.332	0.050
Abnormal thyroid function	0.348	0.036-3.380	0.363

SE, status epilepticus; OR, odds ratio; CI, confidence interval; CNS, central nervous system; ALB, albumin; FT3, free triiodothyronine; TSH, thyroid-stimulating hormone; STESS, status epilepticus severity score.

### The predictive power of FT3 for mortality at 30-day follow up

**Figure 2:**
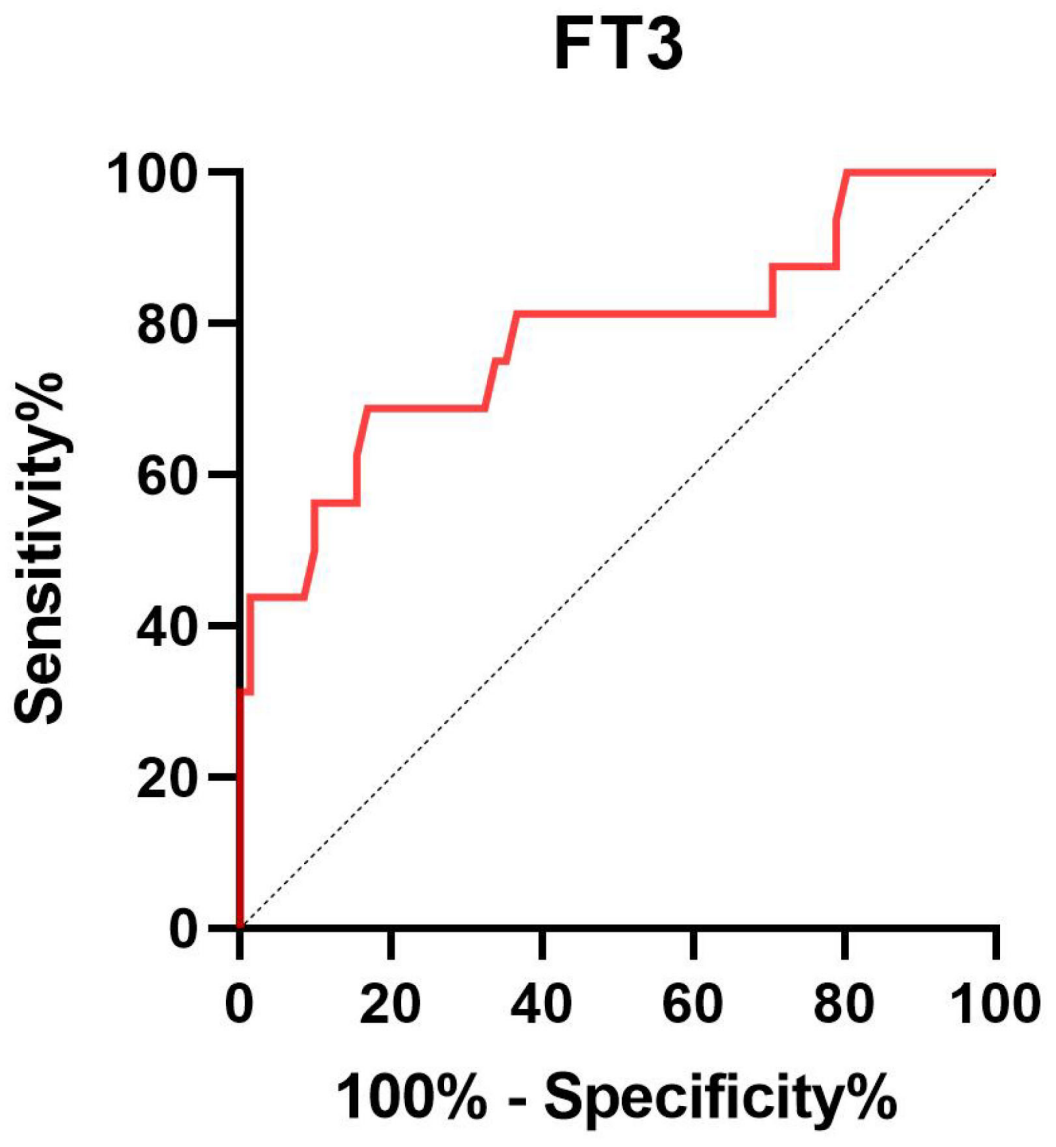
Receiver operating characteristic curve of FT3 to predict 30-day mortality in SE patients. FT3 free triiodothyronine; SE status epilepticus.

Given that FT3 was suggested to be independently related to 30-day mortality, we further carried out the receiver operating characteristic (ROC) curve to evaluate the predictive ability of FT3 for 30-day mortality in SE patients. The area under the ROC curve of FT3 was 0.780 (95% confidence interval: 0.635-0.925, *P* < 0.001) for 30-day mortality (**Figure 2**). The optimal predictive cutoff value for 30-day mortality by FT3 was 1.64 (sensitivity 68.75%, specificity 83.10%).

### Poor outcome at discharge and 30-day mortality stratified by T3 status

The clinical characteristics and outcomes of patients with and without low T3 syndrome were presented in **Table 7**. Poor outcome at discharge was observed more frequently among patients with low T3 syndrome than among patients without low T3 syndrome (*P* < 0.001). Likewise, the proportion of 30-day mortality was remarkably higher in patients with low T3 syndrome than in those without low T3 syndrome (*P* = 0.025).

**Table 7:** Comparisons of outcomes at discharge and 30-day mortality in SE patients with or without low T3 syndrome.

Variable	Low T3 syndrome (n=26)	Without low T3 syndrome (n=61)	*P*
Male (n, %)	12 (46.2)	38 (62.3)	0.163
Age, years, median (IQR)	60 (36-73)	50 (29-59)	0.064
SE etiology grouped according to the ILAE (n, %)			0.118
Acute symptomatic seizures	12 (46.1)	19 (31.1)	
Remote symptomatic unprovoked seizures	4 (15.4)	9 (14.8)	
Symptomatic seizures due to progressive CNS disorders	0 (0)	11 (18.0)	
Seizures of unknown etiology	10 (38.5)	22 (36.1)	
Stroke etiology of SE (n, %)	4 (15.4)	7 (11.5)	0.881
Infectious etiology of SE (n, %)	4 (15.4)	7 (11.5)	0.881
Metabolic etiology of SE (n, %)	1 (3.8)	4 (6.6)	0.995
No history of seizures (n, %)	22 (84.6)	41 (67.2)	0.096
Refractory SE (n, %)	17 (65.4)	28 (45.9)	0.096
Super-refractory SE (n, %)	5 (19.2)	3 (4.9)	0.087
STESS at SE onset, median (IQR)	3 (2-4)	2 (2-2)	0.002
SE duration, days, median (IQR)	3 (1.3-4)	1 (1-2)	0.006
AEDs, median (IQR)	3 (3-4)	3 (2-3)	0.053
Poor outcome at discharge (n, %)	18 (69.2)	13 (21.3)	<0.001
30 day-mortality (n, %)	9 (34.6)	7 (11.5)	0.025

SE, status epilepticus; IQR, interquartile range; ILAE, International League Against Epilepsy; CNS, central nervous system; STESS, status epilepticus severity score; AEDs, antiepileptic drugs; T3 triiodothyronine.

### External cohort validated the predictive value of FT3 for SE prognosis

We further employed an external cohort to validate our findings. Univariable and multivariable analyses performed in the external cohort were displayed in **Tables 8**, **9**, respectively. Univariable analysis identified SE etiology, FT3, albumin, STESS and red cell distribution width as prognostic factors for 30-day mortality in SE patients (all *P* < 0.10, **Table 8**), and these markers were entered into the multivariable analysis (**Table 9**). The multivariate analysis showed that only FT3 was independently associated with 30-day mortality (OR = 0.002; 95% confidence interval, 0.000-0.791; *P* = 0.041). Additionally, SE patients with low T3 syndrome had higher proportion of 30-day mortality compared to those without low T3 syndrome (*P* = 0.013, **Table 10**). Collectively, the results from the external cohort were consistent with the findings of the discovery cohort, which further validated the predictive value of FT3 for the prognosis of SE patients.

**Table 8:** Univariate analysis of clinical characteristics and laboratory data between survivors and non-survivors in the external validation cohort.

Variable	Survivors (n=46)	Non-survivors (n=9)	*P*
Male (n, %)	26 (56.5)	5 (55.6)	0.754
Age, years, median (IQR)	67 (55-77)	60 (57-87)	0.942
SE etiology grouped according to the ILAE (n, %)			0.026
Acute symptomatic seizures	16 (34.8)	5 (55.6)	
Remote symptomatic unprovoked seizures	6 (13.0)	0 (0)	
Symptomatic seizures due to progressive CNS disorders	3 (6.5)	3 (33.3)	
Seizures of unknown etiology	21 (45.7)	1 (11.1)	
Stroke etiology of SE (n, %)	10 (21.7)	3 (33.3)	0.749
Infectious etiology of SE (n, %)	6 (13.0)	2 (22.2)	0.844
Metabolic etiology of SE (n, %)	2 (4.3)	0 (0)	0.737
No history of seizures (n, %)	26 (56.5)	6 (66.7)	0.846
Refractory SE (n, %)	17 (37.0)	5 (55.6)	0.503
Super-refractory SE (n, %)	3 (6.5)	2 (22.2)	0.387
STESS at SE onset, median (IQR)	3 (2-4)	4 (3-5)	0.024
SE duration, days, median (IQR)	1 (1-2)	2 (1-3)	0.186
AEDs, median (IQR)	3 (2-3)	3 (2-3)	0.702
RBC (x10^6^/μL), median (IQR)	4.18 (3.94-4.57)	3.78 (3.15-4.60)	0.342
WBC (x10^3^/μL), median (IQR)	9.06 (6.69-11.06)	7.40 (6.58-9.58)	0.732
NEU (x10^3^/μL), median (IQR)	6.75 (4.73-8.67)	5.93 (4.29-8.89)	0.882
LYM (x10^3^/μL), median (IQR)	1.45 (1.10-1.72)	1.10 (0.96-1.38)	0.170
MON (x10^3^/μL), mean (SD)	0.67 (0.29)	0.76 (0.35)	0.421
PLT (x10^3^/μL), mean (SD)	201 (70)	204 (75)	0.917
RDW (fL), median (IQR)	44.1(42.4-47.0)	46.5 (44.6-49.1)	0.022
ALB (g/L), mean (SD)	39.8 (5.4)	35.1 (9.0)	0.044
Abnormal thyroid function (n, %)	13 (28.3)	5 (55.6)	0.227
FT3 (pg/mL), mean (SD)	1.37 (0.27)	0.98 (0.32)	<0.001
FT4 (ng/dL), median (IQR)	0.93 (0.80-1.06)	0.73 (0.55-1.02)	0.116
TSH (mIU/L), median (IQR)	2.32 (1.67-3.22)	3.16 (1.71-6.91)	0.340

SE, status epilepticus; IQR, interquartile range; ILAE, International League Against Epilepsy; CNS, central nervous system; STESS, status epilepticus severity score; AEDs, antiepileptic drugs; SD, standard deviation; RBC, red blood cell count; RDW, red cell distribution width; WBC, white blood cell count; NEU, neutrophil count; LYM, lymphocyte count; MON, monocyte count; PLT, platelet count; ALB, albumin; FT4, free thyroxin; FT3, free triiodothyronine; TSH, thyroid-stimulating hormone.

**Table 9:** Multivariate analysis of predictors for 30-day mortality in SE patients in the external validation cohort.

Variable	OR	95% CI	*P*
SE etiology			
Acute symptomatic seizures	Reference	–	–
Remote symptomatic unprovoked seizures	0.000	0.000	0.999
Symptomatic seizures due to progressive CNS disorders	35.528	0.655-1926.159	0.080
Seizures of unknown etiology	0.320	0.014-7.180	0.473
FT3	0.002	0.000-0.791	0.041
ALB	1.018	0.817-1.268	0.876
STESS	2.785	0.756-10.253	0.124
RDW	1.052	0.854-1.296	0.634

SE, status epilepticus; OR, odds ratio; CI, confidence interval; CNS, central nervous system; ALB, albumin; FT3, free triiodothyronine; STESS, status epilepticus severity score; RDW, red cell distribution width.

**Table 10:** Comparisons of SE outcomes in patients with or without low T3 syndrome in the external validation cohort.

Variable	Low T3 syndrome (n=15)	Without low T3 syndrome (n=40)	*P*
Male (n, %)	7 (46.7)	24 (60.0)	0.375
Age, years, median (IQR)	67 (57-81)	67 (55-77)	0.691
SE etiology grouped according to the ILAE (n, %)			0.951
Acute symptomatic seizures	5 (33.4)	16 (40.0)	
Remote symptomatic unprovoked seizures	2 (13.3)	4 (10.0)	
Symptomatic seizures due to progressive CNS disorders	2 (13.3)	4 (10.0)	
Seizures of unknown etiology	6 (40.0)	16 (40.0)	
Stroke etiology of SE (n, %)	2 (13.3)	11 (27.5)	0.456
Infectious etiology of SE (n, %)	3 (20.0)	5 (12.5)	0.785
Metabolic etiology of SE (n, %)	0 (0)	2 (5.0)	0.941
No history of seizures (n, %)	8 (53.3)	24 (60.0)	0.655
Refractory SE (n, %)	10 (66.7)	12 (30.0)	0.013
Super-refractory SE (n, %)	3 (20.0)	2 (5.0)	0.231
STESS at SE onset, median (IQR)	3 (3-4)	3 (2-4)	0.061
SE duration, days, median (IQR)	2 (1-3)	1 (1-2)	0.097
AEDs, median (IQR)	3 (3-4)	2 (2-3)	0.008
Poor outcome at discharge (n, %)	11 (73.3)	10 (25.0)	0.001
30 day-mortality (n, %)	6 (40.0)	3 (7.5)	0.013

SE, status epilepticus; IQR, interquartile range; ILAE, International League Against Epilepsy; CNS, central nervous system; STESS, status epilepticus severity score; AEDs, antiepileptic drugs; T3, triiodothyronine.

## Discussion

Our study indicated that serum levels of FT3 measured on admission were independently associated with 30-day mortality in SE patients. Our findings supported that serum FT3 level was an independent predictor for the prognosis of SE patients. Additionally, we also observed that low T3 syndrome was associated with poor outcome at hospital discharge and higher risk of mortality in SE patients. Of note, this study for the first time explored the correlation between thyroid hormones and SE prognosis.

Thyroid hormones, including T3 and T4, are synthesized and released by the thyroid gland and play a vital role in brain development as well as the maintenance of brain function (18). Thyroid hormone disorders can lead to dysregulated energy metabolism of the central nervous system (CNS), further destroy biological processes including survival and differentiation of neurons, cytoskeleton dynamics and neurotransmission, etc. (19). Thyroid hormones have been suggested to be involved in the pathogenesis of epilepsy. Thyroid hormones exert genomic and non-genomic effects on mitochondrial biogenesis and function, and the decreased activity of thyroid hormones has been indicated to be associated with mitochondrial dysfunction as well as the resulting oxidative stress, thus promoting the generation of epileptic seizures and the development of chronic epilepsy (11). Moreover, thyroid hormones can regulate the development and function of GABAergic neurons (12, 20). As we know, GABA, as the principal inhibitory neurotransmitter, exerts a crucial role in seizure suppression (21). Therefore, it can be speculated that thyroid hormone abnormalities may result in dysregulation of GABAergic system, further disrupting the excitatory-inhibitory balance, ultimately triggering seizures. Of note, the association of thyroid function with epilepsy might be not causal. A recent study applied a Mendelian randomization analysis to investigate the potential causal relationships between thyroid disorders and various types of epilepsy, which indicated no significant causal relationships therein (22). Actually, a bidirectional interplay may exist between thyroid hormones and seizure. In addition to the involvement of thyroid hormones in epileptogenesis as mentioned above, seizure could also result in the variations in thyroid hormone levels, which may be involved in its impact on the hypothalamic-pituitary-thyroid axis. Neuronal hyperactivity during a seizure activates the hypothalamus by specific neurotransmitter alterations or by the release of other mediators, and thus affects the secretion of thyrotropin releasing hormone (TRH), further modulating TSH secretion as well as subsequent thyroid hormone biosynthesis and secretion (23). Thus, it seems that the correlation between thyroid hormones and seizure is complex and diverse.

Despite that the potential relationship between thyroid hormones and seizure has been proposed, studies on thyroid hormones and prognosis of seizure are rare. Our study for the first time investigated the predictive value of thyroid hormones for SE prognosis. We first observed that serum FT3 levels were remarkably decreased as the SE severity increased, and patients with super-refractory SE had the lowest FT3 levels, which indicated the potential relationship between thyroid hormones and SE outcome. Furthermore, our results revealed that serum FT3 level was an independent predictor for 30-day mortality in SE patients. It is suggested that T3 could promote astrocytic glutamate uptake and thus protect astrocytes and neurons against glutamate toxicity (24), which may explain the correlation between low serum FT3 levels and poor SE prognosis. Similar to our results, lower serum FT3 levels have been found to be associated with poor prognosis of multiple CNS diseases. The study by O’Keefe et al. (25) revealed that lower FT3 levels were related to worse outcomes after ischemic stroke. Another investigation indicated that declined FT3 levels as well as increased TSH levels were associated with worse functional outcome and higher risk of mortality within 6 months after traumatic brain injury (13). However, several studies have shown a different result. The research by Ray et al. (26) investigated TSH, T3, and FT4 levels in 180 seriously ill patients after 3 hours of ICU admission, which demonstrated no statistically significant differences in T3 and FT4 levels between survivors and non-survivors. The possible explanations for the inconsistent results may be different disease backgrounds, the differences in the timing of blood sample collecting and the methods of thyroid hormones tested, etc.

Low T3 syndrome is characterized by declined serum T3, decreased or normal serum T4, and normal TSH levels, which has been reported to be closely associated with poor functional outcomes, unfavorable prognosis, and higher risk of mortality in serious diseases (27, 28). In our study, we observed that SE patients with low T3 syndrome had poor outcome at discharge and higher 30-day mortality. Critical diseases cause a declined peripheral conversion of FT4 to FT3, which may lead to the development of low T3 syndrome (29). Therefore, we presumed that SE patients with low T3 syndrome may have relatively severe disease states, and thus suffer from worse outcome. Although low T3 syndrome is reported to be associated with adverse outcomes of various severe acute and chronic diseases, it is controversial whether patients with serious illness accompanied by low T3 syndrome can benefit from thyroid hormone supplementation in the acute stage of diseases, since a decreased serum T3 level may be a compensatory mechanism to conserve energy and diminish protein consumption (30).

Of note, long-term treatment with AEDs has been reported to be associated with changes in thyroid hormone metabolism. The underlying mechanism remains unclear, which may be that AEDs affect hepatic microsomal enzyme systems or uridine diphosphate glucuronosyltransferase, and thus interfere the metabolism of thyroid hormones (31). Several previous studies investigated the effects of different AEDs on thyroid hormones. Adhimoolam et al. (32) compared thyroid hormone levels between adult epileptic patients receiving conventional or newer AEDs and healthy adults, and they found that epileptic patients receiving conventional AEDs such as sodium valproate, carbamazepine and phenytoin had significantly lower FT4 and TSH levels, while no significant difference in thyroid hormones was observed between epileptic patients treated with newer AEDs and healthy adults. Thus, they proposed that conventional AEDs may have remarkable alteration in the thyroid hormone levels compared to the newer AEDs. However, a recent meta-analysis from Han et al. (33) indicated that both conventional and newer AEDs could affect thyroid function. Their results suggested that conventional AEDs including carbamazepine and phenytoin were closely associated with decreased T4 and T3 levels, and newer AEDs like topiramate may result in elevated TSH levels. Additionally, both levetiracetam and valproic acid may lead to subclinical hypothyroidism. In our study, since most of enrolled patients received sodium valproate, we did not evaluate and compare the effects of conventional and newer AEDs on thyroid hormones as well as on the association between thyroid hormones and SE prognosis, and future studies are needed to explore this issue.

Although our results have shed light on the value of thyroid hormones, particularly FT3 for the prediction of SE prognosis, there are some limitations in our study. First, despite that this study included two independent cohorts, each cohort had a relatively small sample size. Second, the blood samples detected in our study were obtained within 24 hours after admission, and dynamically detecting changes in thyroid hormone levels is lacking. Third, some AEDs could affect the function of thyroid hormones, and our study only compared the difference in the number of AEDs between SE patients with good outcome and poor outcome, which indicated no significant differences therein. However, the present study did not yet assess the impact of different types of AEDs, and future investigations are needed to clarify whether various AEDs could affect the correlation between thyroid hormones and SE prognosis. Fourth, the current study only assessed SE outcome at discharge and 30 days after SE onset, future studies are needed to explore the association between thyroid hormone levels and the long-term prognosis of SE.

## Conclusions

Lower serum FT3 level was independently associated with 30-day mortality in SE patients. Low T3 syndrome could be a potential biomarker for predicting the prognosis of SE. Future large sample, multi-center, prospective studies are needed to validate our results and to evaluate the potential of thyroid function and low T3 syndrome as reliable markers for predicting the long-term prognosis of SE.

## Data Availability

The original contributions presented in the study are included in the article/supplementary material, further inquiries can be directed to the corresponding author/s.
